# The four main domains of the concept of return-on-investment from healthcare quality improvement programmes

**DOI:** 10.1093/intqhc/mzag089

**Published:** 2026-06-18

**Authors:** S’thembile Thusini, Tayana Soukup, Claire Henderson

**Affiliations:** Health Services and Population Research Department, King’s College London, Institute of Psychiatry, Psychology and Neuroscience, London, SE5 8AF, United Kingdom; Imperial College London, Department of Surgery and Cancer, London, W12 0NN, United Kingdom; Centre for Implementation Science, King’s College London, London, SE5 8AF, United Kingdom

**Keywords:** Healthcare, Quality Improvement, QI, Return on Investment, ROI

## Abstract

**Background:**

This paper synthesizes a series of four studies in the exploration of the concept of Return on Investment (ROI) in relation to healthcare Quality Improvement (QI) programmes (QI-ROI).

**Methods:**

We followed a multi-stage mixed-methods integration approach where four studies were first conducted and reported separately. The four study designs were linked to enable us to build a cohesive understanding of QI-ROI. Collective findings were then analysed and interpreted to identify the main QI-ROI concept domains. Two studies were based on an extensive global interdisciplinary systematic literature review *N* = 68; one a qualitative study *N* = 16, and last, a Delphi study *N* = 23. For the latter two studies, participants were from the UK National Health Service and included 24 board members, 15 service and clinical directors, and QI leaders.

**Results:**

Several benefits were seen to represent ROI from QI programmes. These encompassed internal (e.g. development) and external (e.g. service user socio-economic benefits), intervention outcomes (e.g. clinical benefits) and implementation outcomes (e.g. spread). Further, the benefits included lessons from ‘failed’ QI programmes. Together, these benefits encompass monetary and non-monetary value of QI along a programme’s journey. We view these benefits as four main domains: development, improvement, savings, and sustainability or DISS.

**Conclusion:**

QI has several organizational and health system benefits contained within the DISS construct. These benefits support various organizational goals such as quality improvement, organizational development, performance and resilience. We posit that the collective findings are applicable to many organizations that provide interdisciplinary healthcare services globally.

## Background

Healthcare organizations face multiple budgetary demands in the care of their populations. Consequently, leaders must make informed decisions about where best to allocate limited healthcare funds, thereby limiting opportunity costs. Fund allocation decisions occur at different levels including global, national, and organizational levels. To this effect, Return on Investment (ROI) is increasingly encouraged as a tool to develop business cases that motivate for initial or continued investment [1, 2]. This includes funding of Quality Improvement (QI) programmes [3, 4]. Large-scale QI programmes use various methods to improve healthcare quality and safety from an organizational level to health system-wide collaboratives [5].

We recently completed serial evaluations on the concept of ROI from large QI programmes to develop a QI-ROI conceptual framework. We referred to return-on-investment and QI-ROI to denote our study of ROI as a concept and not a metric. Our focus was perceptions of QI-ROI, the evidence of which will require future research. Our work was framed from the context of the United Kingdom (UK) National Health Service mental healthcare (NHS), a publicly-funded institution which provides acute and long-term care from communities to hospitals. The factors that determined QI-ROI concept may be shared by similar organizations globally, where our findings may also apply. These determinants are discussed in depth elsewhere [6].

## Materials and methods

Our research followed a sequential confirmatory-exploratory-explanatory design guided by Fetters *et al.* [7]. This multi-staged approach included connecting, building, and embedding [7]. Through embedding, we connected our research project at multiple points; from informing questions to be addressed next, then purposively sampling participants and deductive-inductive analysis. Further, we exercised abduction and retroduction [8]; i.e. we analysed and interpreted new insights to describe the QI-ROI concept. As such, results of successive studies served to confirm previous knowledge, as well as explore and explain new insights through the building process. To report our results, we used a ‘joint display’ [7] of findings from each study to illustrate how each added to our conclusions on the four main QI-ROI domains.

To identify the main QI-ROI domains we also adopted Sartori’s approach to concept formation where our research focused on naming ‘what (QI-ROI) is’ [9]. This involved identifying the various ‘more-or-less’ [9] attributes and categories contained within QI-ROI as articulated by authors and participants across our studies. We thus avoided the ‘either-or’ [9] question, thereby enabling ambiguity tolerance across our studies. We now ‘weave’ [7] our findings to identify the main domains of the QI-ROI concept. This paper reports on this synthesis.

### Synthesis summary

The UK mental healthcare is where the question about QI-ROI arose and thus the main setting for our research. However, we started with a two-legged global multidisciplinary systematic literature review [10, 11], followed by qualitative interviews [12], and a Delphi [13]. The last two studies included leaders from UK’s NHS mental healthcare: board members (*n* = 24), clinical, service, and QI leaders (*n* = 15). Board members included chief, operational, financial, and QI executives. The governing boards of directors were leaders deemed accountable for funding QI programmes, and thus ‘QI investors’. Some represented staff (medical, nursing, allied) and service-users (non-executives). Senior clinical, service, and QI leaders outside the board were deemed influential in QI related matters. We first present a summary of study 1.

### Study 1

In this literature review (*N* = 68), we analysed and developed the QI-ROI concept [10]. We noted that ‘cost’ was largely used to denote investment, whilst the concepts to denote returns included value, benefit, cost-saving, profit, development, improvement, effectiveness, and efficiency. Terms used to denote returns v investment included Cost-Effectiveness Analysis, Cost-Benefit Analysis, and Social ROI. These ROI-like concepts are discussed in detail here [10]. Briefly, we viewed these concepts as part of QI-ROI at different levels. Some represented inputs versus outputs, outcomes or impacts, e.g. staff may be up-skilled (development), resulting in efficiency (output), improving quality, costs (outcomes), and revenue (impact). All these are beneficial but only have perceived value if perceived to have utility.

### Study 2

In this leg of our systematic literature review [11], we expanded on the conceptual framework by exploring the benefits and relationships within the domains of the QI-ROI concept. We noted four themes: (i) organizational performance (e.g. patient and financial outcomes), (ii) organizational development (e.g. capacity and capability), (iii) external outcomes (e.g. socio-economic benefits), and (iv) unintended outcomes (positive/negative). Authors reported positive aspects of negative unintended consequences (e.g. lessons). Benefits appeared interlinked, and ROI from QI was not merely an end-outcome; earlier benefits such as efficiency were seen as a form of ROI in and of themselves. This indicated a contemporaneous hierarchy of QI-ROI.

### Study 3

This study [12] explored the findings from the global literature through interviews in one UK NHS mental health organization (*N* = 16). Here, QI-ROI was also conceptualized as any valued monetary or non-monetary benefit where QI contributes to an overall organizational strategy. Valued benefits included benefits for patients, staff, external partners, and implementation outcomes such as sustainability. The importance of financial benefits was raised, albeit with apprehension over monetization. Lessons from failed QI were also valued. We noted potential QI-ROI concept instability linked to multiple goals and outcome uncertainty. However, health and social care values supported desires for comprehensive QI-ROI for multiple stakeholders. Further socio-political desires to improve quality drove continued QI investment.

### Study 4

This study [13], explored ambiguity and consensus on the QI-ROI concept with mental health leaders from multiple UK sites (*N* = 23). The two round Delphi indicated consensus on 45 of 67 (67%) and dissensus on 22 of 67 (33%) items. Patient outcomes were rated as most relevant, followed by development and benefits for external stakeholders. External incentives (e.g. competitiveness) and monetized outcomes were rated as least relevant or legitimate. Indecision or dissensus were largely on implementation outcomes, timing, measurement and monetization of benefits. Qualitative data indicated that indecision and dissensus were linked to ambiguity and uncertainty, related to local needs or novelty of some benefits locally e.g. profit and status. This study consolidated our understanding of the ‘more-or-less’ QI-ROI attributes.

### Synthesis

Across studies, internal benefits such as health outcomes and patient experience were seen as the primary benefits sought from QI. Development of various aspects of an organization including staff, culture, and sustainability were also highly valued. Further, external benefits e.g. service user socio-economic benefits and external collaboration were valued. Though not primary, cost-saving, cost-avoidance, and financial sustainability appeared to be of increasing significance. However, this was set against concerns over benefit monetization as a reflection on traditional ROI against the goals and expectations from healthcare QI. Thus, ambiguity and uncertainty over certain QI benefits appeared inherent to QI-ROI conceptualization.

**Figure 1:**
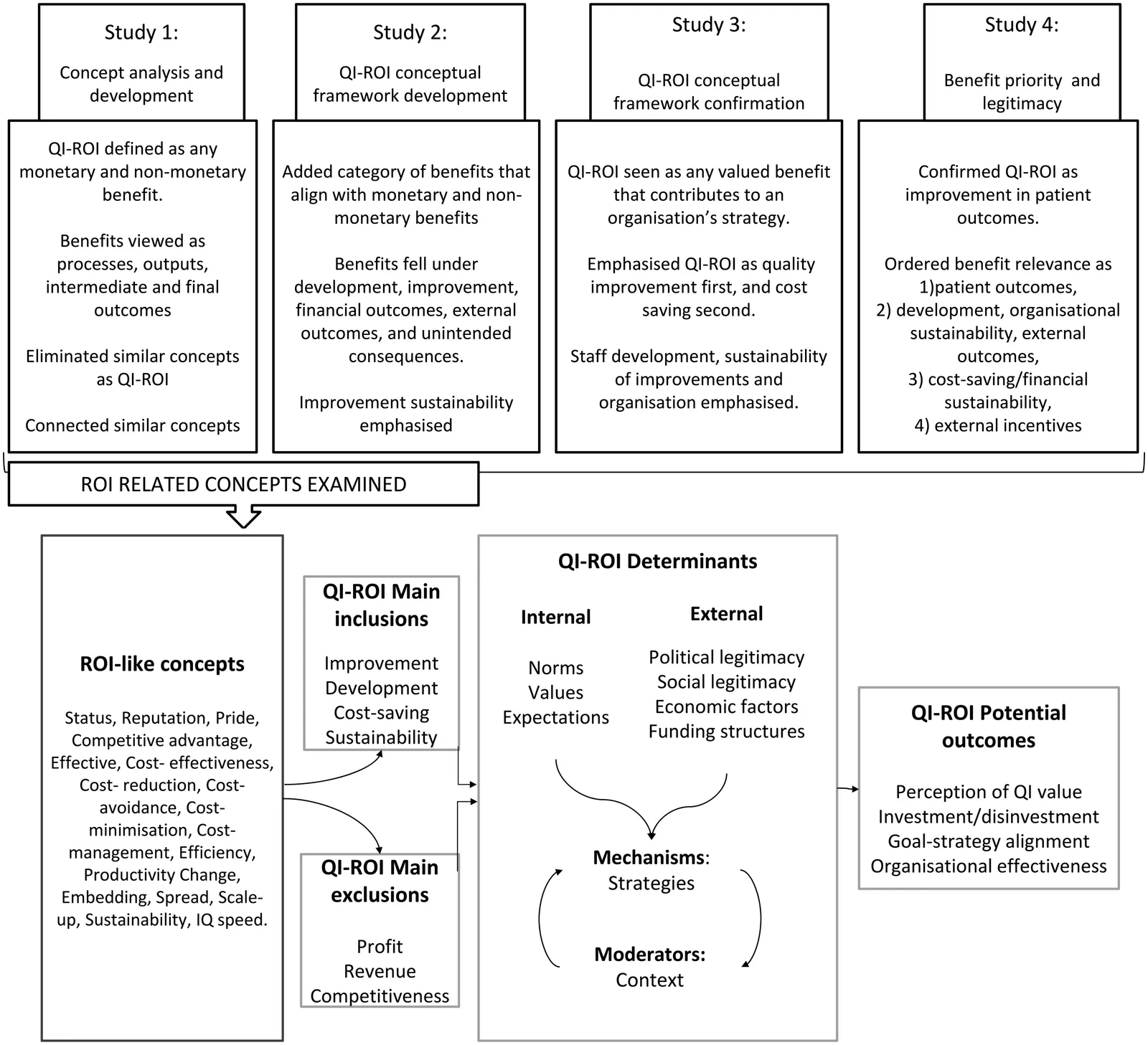
The development of the QI-ROI concept. Top half: Goals and conclusions from each study. Bottom half: ROI-like concepts explored. Bottom half from left are all the prevalent terms and concepts found to be associated with ROI from QI across studies. These were later divided into the concepts deemed to strongly represent QI-ROI (inclusions) and those less associated with QI-ROI (exclusions). As per Morse et al. [13], we also included potential determinants and outcomes of this QI-ROI conceptualization. We note that different determinants can lead to addition of new or reprioritization of existing benefits. These changes may not change the main QI-ROI domains (DISS), but create internal dynamism, i.e. reconfigure the position of certain benefits under the overall DISS framework. However, DISS extensions may be possible if entirely new domains emerge in the future.

Our findings indicated that the main attributes may remain, but the QI-ROI concept is dynamic. Nonetheless, Morse *et al.* [14] stated that ‘a mature concept should be well defined, with attributes identified, boundaries demarcated, preconditions specified, and outcomes described’ (p. 255). In Figure 1, we begin this process by describing the QI-ROI concept as we see it currently. We include (i) concepts examined, (ii) potential determinants (preconditions), (iii) and concept outcomes (e.g. investment). Determinants include internal factors (e.g. beliefs) and external factors (e.g. funding structures) that influence QI-ROI conceptualization as monetary or not, which could lead to outcomes such as investment or dis-investment. Figure 1 includes a joint display of findings that contributed to the identification of the four main domains.

Notably, leaders appeared unlikely to disinvest due to beliefs that QI was intrinsically linked organizational goals and values [12]. Rather, leaders sought to re-evaluate QI strategies, noting valuable lessons as part of development. Thus, internal factors (e.g. beliefs) or external factors (e.g. funding) may be executed through QI strategies but moderated by contexts to yield certain ROI conceptualizations. These may be unique to context or time, e.g. intraorganizational programmes, collaboratives and highly developed organizations may have different conceptual determinants, boundaries and outcomes. This may add new or prioritize other benefits.

The UK public healthcare appeared to adopt a patient and staff focus, from local to systems perspectives. Novel benefits such as profit, status or competitive advantage appeared to have little or no role in conceptualizing QI-ROI. If gained, these may be considered incidental. However, being provider of choice and competition have been linked to financial incentives in the UK NHS. Further, cost-savings and revenue generation were viewed as mechanisms for improving care [12]. As such, QI-ROI contains attributes of intrinsic value (a benefit of value in itself) and extrinsic or instrumental value which leads to attainment of intrinsic value [15].

**Figure 2:**
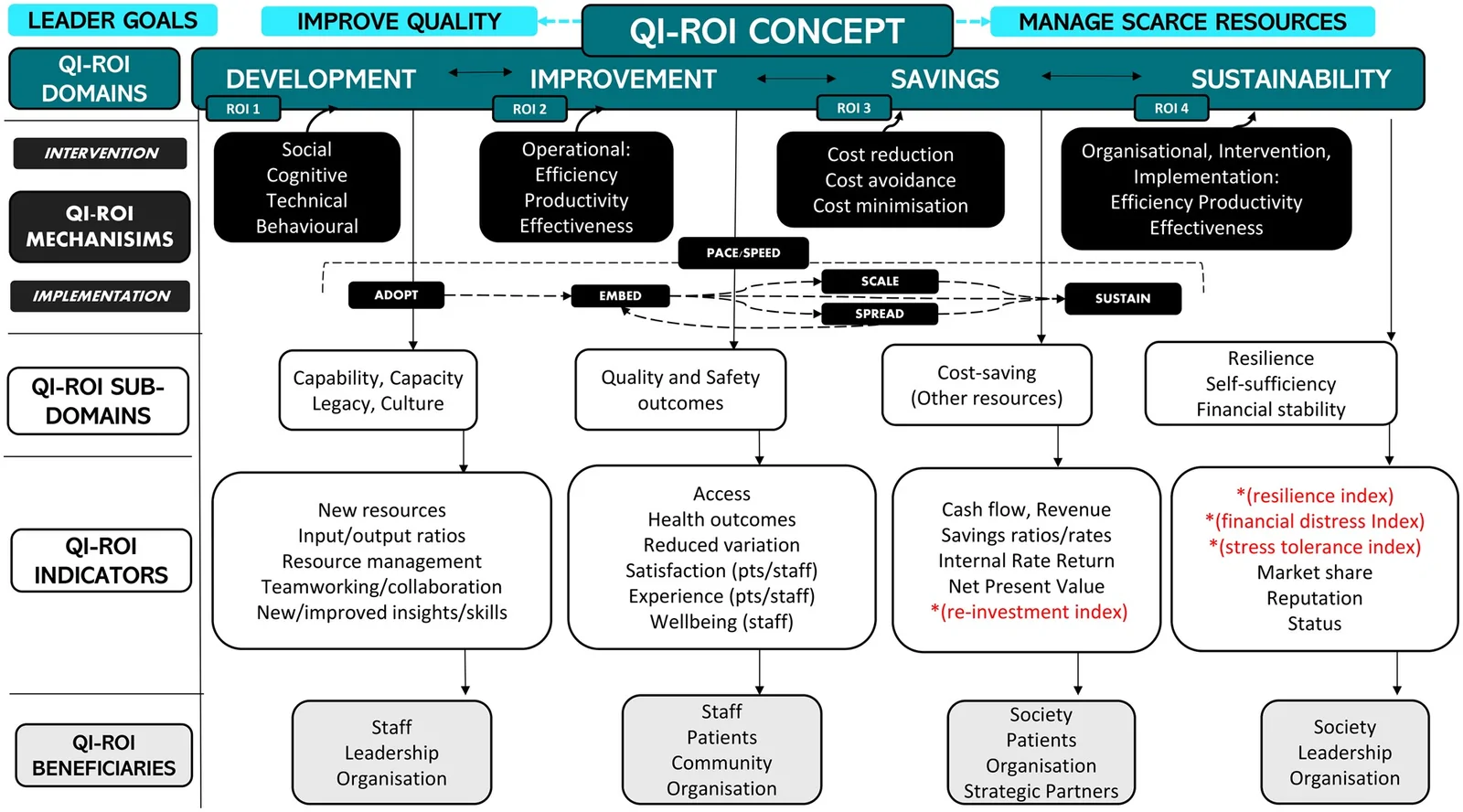
The DISS domains, subdomains and indicators. Figure 2 illustrates the DISS framework and its components. In light blue, are the leaders’ overriding determinants for the presented QI-ROI conceptualization. In deeper blue, are the four main domains of the QI-ROI concept that connect throughout a QI programme lifetime. The connections may be nonlinear with different start-points, depending on development level and other contextual configurations. Below, the main domains connect to their sub-domains, indicators, and potential beneficiaries. We include potential data sources that can support QI-ROI evaluation. In black, are intervention and implementation mechanisms that connect DISS domains overtime across a QI journey. *Suggested measures: [to our knowledge] the ‘re-investment index’ and ‘financial distress index’ do not exist, but could be developed or borrowed from other industries, e.g. resilience indicators.

To illustrate QI-ROI attributes, Fig. 2 presents a web of multi-level QI benefits within the QI-ROI ‘ladder of abstraction’ [9], e.g. efficiency sits lower to development, which sits lower to QI-ROI. As seen here, the QI benefits articulated fell into four main functions represented by concepts; develop, improve, save, sustain. Thus, we concluded that the four main QI-ROI domains are: Development, Improvement, Savings, and Sustainability; acronym DISS. These domains follow four main phases in a QI programme’s journey from immediate benefits to impacts. Development is often the first benefit or set of benefits and therefore ROI 1, followed by improvement (ROI 2), savings (ROI 3), and sustainability (ROI 4).

DISS domains feed to each other across a temporal (horizontal) plane to help balance the organizational needs to improve quality and manage scarce resources over time. Vertically, the domains connect to their more observable parts [16] e.g. development (domain), capability (sub-domain), cognitive change (mechanism), new skill (indicator). These reflect QI-ROI’s ‘ladder of abstraction’ from the frontline to the board level. Abstract indicators like patient experience can be isolated at the programme level or presented as composite measures of overall QI value.

Three stakeholder levels of QI-ROI are thus apparent: micro (frontline), meso (organization), and macro (system/society). This aligns with Satori’s differentiation of concepts as low-level (applied locally), mid-level (across regions), or high-level (national or international). The DISS framework is thus a ‘data container’ [9] of QI benefit clusters. Some of these may not be directly related or causal, but parallel, e.g. development may consist of staff and process development, both with efficiency as an indicator. DISS can also be viewed as ‘mid-range theorisation’ [9] linking QI value to traditional ROI through contextualized low-level concepts.

The QI benefits discussed indicate various dimensions, e.g. (i) internal benefits (e.g. culture) and external benefits (e.g. socio-economic); (ii) intervention benefits (e.g. clinical) or implementation benefits (e.g. scale). Notably, monetary and non-monetary QI-ROI appeared co-dependant. For example, developing capacities to improve and successful implementation (e.g. scaling up) may be a mechanism for efficient QI use. Both could lead to cost-saving, which supports health outcomes. We see these as interlinked components of QI-ROI. We imagine that the connections are not linear. For example, developed organizations with sustained improvements could simultaneously enjoy savings, improvements, and development.

In Fig. 2, some measures (in red) are conceptual as a way to imagine the operationalization of this QI-ROI concept, subject to future research. Implementation outcomes like embed and spread are illustrated as mechanisms that connect DISS domains, e.g. adoption requires development before improvement, which may later lead to savings and sustainability. To our knowledge, no measure exists to link QI savings to financial or organizational sustainability. Thus, we suggest that metrics such as a form of ‘re-investment index’ may illuminate QI contributions. Further, a ‘financial distress or resilience index’ may help indicate sustainability.

Ultimately, largely similar QI-ROI attributes were observed across our studies. Patient outcomes were central, other benefits supported this goal. Some benefits had desired utility and thus valued. The valuing of benefits in a more-less fashion suggests that QI-ROI dynamism may largely be internal, within the framework. We concluded that QI-ROI is conceptualized as any valued benefit based on context. The benefits need not compete and can be synergistic.

## Discussion

The purpose of this paper was to synthesize the findings from our research on ROI from QI to highlight the main domains of the QI-ROI concept in the UK mental healthcare context and similar organizations. Our first two studies drew data from global literature, whilst the last two were UK focused. We concluded that QI-ROI has four umbrella domains: development, improvement, savings and sustainability; DISS. Together, these benefits encompass monetary and non-monetary value along a programme’s journey. The DISS includes lessons from ‘failed’ QI that help organizations develop and improve. Within these, organizations may select or add nuanced benefits depending on their needs and goals. Our QI-ROI concept definition as any valued benefits is broad, but intentionally so, to allow adaptability and further development.

Accepting conceptual ambiguity seems paradoxical in a study seeking conceptual clarity. However, some argue that ambiguity is not a defect, but a necessary trait that makes concepts useable [9, 17]. Berenskoetter [17] asserted that concepts are ‘broad and complex … plural, shifting, and incomplete …’ (p. 5). However, to minimize ‘epistemological anarchy’ [18] flexibility must be balanced with specificity that enables ‘operational meaning’, with minimal loss of ‘conceptual meaning’ [9]. In this regard, the QI-ROI concept in the form DISS domains allows for contextualization of QI-ROI, making it focused and meaningful in context and time.

The DISS forms what Satori [9] referred to as the ‘ideal type’ concept, with improvement as the ‘core trait or intension concept’, and development, savings, and sustainability as ‘extension concepts’. Any benefit that contributes to an organization’s goals within one of these domains represents QI-ROI in that context. QI-ROI as defined here is viewed as a process, not an event. An event implies a dichotomy, e.g. either improvement exists or it doesn’t. A process implies broad temporal benefits. QI-ROI thus is viewed as contemporaneous and hierarchical.

Developmental factors such as culture are often seen as improvement preconditions rather than QI outcomes [19]. However, development can occur intentionally or unintentionally as part of a QI implementation strategy. Improving health outcomes often requires improved processes, systems, and behaviours. QI as a bottom-up approach requires staff engagement. As such, QI programmes can support the psychological safety needed for staff and teams to develop required capabilities and capacities, as well as lead to new relationships. Thus, current QI improves adoption, embedding, and sustaining future QI’s. Staff development can improve effectiveness, efficiency, productivity, savings, sustainability, and organizational development. These can sustain positive cultures and impact system-level outcomes such as resilience [20].

Development enables improvement. Improvement implies a change from an undesired to a desired, or more desirable state, as demonstrated by the achievement of measurable goals, and linked to evidence-based care. QI effectiveness has been viewed as synonymous to ROI [4]. In ROI, effectiveness means measurable monetized returns [4]. However, in large-scale QI, effectiveness appears broader. An internationally accepted description of large QI describes them as comprehensive interventions by and for multiple stakeholders, aimed at to improving abilities to efficiently identify and innovatively solve problems, and strengthen systems [21].

This description fits with systemic quality, and an appropriate view of effectiveness should include overall QI success. We see QI success as encompassing intended and unintended benefits from both effectiveness and failure. This is steeped in the QI trial-and-error philosophy where failure is success if it leads to valuable lessons [12]. Failure includes intervention failure (goal not achieved) and implementation failure (e.g. not sustained). Failure is also be linked to negative outcomes like blame. QI failure may thus reflect systemic failure. As such, from large QI perspective, a narrow ROI view may miss systemic benefits that contribute to savings.

Within financial outcomes, cost-saving was the second benefit most strongly associated with QI-ROI, after financial sustainability. Cost-savings can result from efficiency, avoiding and or reducing costs [10]. Swensen *et al.* [3] stated that the main source of ROI is removal of waste. Programmes such as ‘Choosing Wisely’ target such efficiency savings [22]. Savings lead to retained funds from sales or allocation (revenue) rather than excess gained (profit). In for-profit contexts savings may be deemed profit. In public healthcare, revenue is generated from funding streams, incentives or adding private practices. In the UK, leaders seek to re-invest retained funds (savings) into organizations to support health outcomes and sustainability.

In Implementation Science, Proctor *et al.* [23] defined sustainability as ‘the extent to which a newly implemented treatment is maintained within … stable operations’ (p. 70). Sustainability is a long-term outcome. Earlier QI stages test feasibility, appropriateness, and acceptability of interventions [23]. Findings may be used to adapt interventions, support adoption or fidelity in context [23]. Financial sustainability refers to the continued ability to independently fund service provision using savings from various sources [24]. Financial sustainability and programmatic sustainability are co-dependant; finances sustain QI programmes; sustained programmes support financial sustainability. Together, they can sustain organizations.

At an organizational level, sustainability is the capacity and capability to permanently meet the needs of stakeholders [25]. The concept of organizational sustainability in healthcare appears undeveloped. However, sustained finances, intervention and implementation outcomes may be part of this construct. In our studies, we noted that abilities to identify and solve problems fast is linked to sustainability [12, 13]. Viewing sustainability as a process, rather than an end may enable insights into organizational stresses threatening sustained quality care. Indices such as the suggested ‘re-investment index,’ ‘financial distress or resilience index’ may be useful. Chen et al. [26] recently developed resilience measures using case studies from commerce (e.g. Apple and Microsoft). These may guide the development of healthcare resilience measures.

QI-ROI conceptualized as the DISS domains thus supports overall healthcare systems functions such as organizational development, effectiveness, efficiency, and performance. This includes building adaptable, collaborative and integrated health systems, and connects healthcare outputs with socio-economic and global priorities [27]. In this regard, the DISS framework may facilitate collaboration by supporting inclusion of externalized outcomes. Similar to the Balanced Score Card [28], DISS may support ambiguity and uncertainty management by QI and organizational leaders through promotion of a balanced view of ROI from QI.

Notably, the DISS framework excludes traditional ROI concepts such as profit and net-present-value. These may be valued in other contexts, and may be added as extensions or specified within DISS. However, we suggest that benefits such as revenue, profit, status, competitiveness and reputation result from achieving DISS, partly or fully. The DISS configuration avoids the often-futile quests for monetization [29], which may support ROI operationalization in healthcare QI, whilst aligning with local philosophies.

Good governance may mean learning and borrowing business ideas such as ROI. However, uncritical spreading of such risks negative consequences for organizations, health systems and societies, e.g. ‘public value failure’ [30], and unhelpful competition or inefficiencies seen during the COVID-19 pandemic [31]. Therefore, healthcare governance must adequately account for both subjective and objective value. The DISS framework could enable leaders to re-imagine QI value and related concepts such as effectiveness and consider external and non-monetary benefits as legitimate benefits parts of ROI, as well as the interconnected elements needed for overall success. Crucially, DISS highlights effective and efficient implementation as part of QI-ROI.

The overriding determinants for the described QI-ROI concept were needs to improve quality whilst managing scarce resources, with highest value placed on patient outcomes. Both non-profit and for-profit health systems grapple with this challenge. Addressing complex systemic quality and value-based care requires collaboration, continuous improvement and adaptability. Unless resolving healthcare misuse, non-monetary benefits likely precede financial benefits. Highly developed organizations may realize monetary ROI faster. At any developmental level, the DISS framework dynamism allows contextualization across a QI journey.

## Limitations

Our studies largely focused on mental healthcare organizations in the publicly-funded UK system. However, our systematic review took a global perspective. We acknowledge that other QI-ROI interpretations are possible, even within the UK, e.g. patients, staff and commissioners’ perspectives. These perspectives were not directly engaged in our studies due to our focus on organizational-level QI investors. Additionally, it is worth noting that including immeasurable and non-monetizable benefits legitimizes subjectivity and avoids intractable monetization but brings inherent challenges such as ambiguity and uncertainty.

## Implications

### Research

Measuring QI-ROI will require innovation, e.g. tracking and linking QI savings. Firstly, the championing of non-monetary benefits challenges evaluators to meaningfully incorporate these in the valuation of QI-ROI. Secondly, our research has implications for capturing QI benefits traditionally deemed as externalized benefits. Evidence-based tools would help manage QI-ROI ambiguities and uncertainties. Lessons from practices such as SROI and multicriteria decision analysis may help develop measures that capture benefits within the DISS domains. Thirdly, although our findings championed patient and staff benefits, direct engagement with these stakeholders in future researcher may deepen understanding of QI-ROI. Lastly, we found that implementation outcomes matter for QI effectiveness, efficiency and sustainability. Implementation science tools may help collect data that can be used to assess the value of QI.

### Practice

QI-ROI in the form of DISS may support various QI and organizational aspects. For example, QI leaders may better discern and articulate QI value to QI investors, QI investors may be able to better account for QI investment, better decisions could then be made about where and how best to use QI and organizational development investments, as well as where and how best to measure value. These aspects may help manage expectations about QI value moving forward, thereby facilitating productive stakeholder engagement to better align QI to organizational strategies. In this way, the DISS framework can serve as a QI-ROI menu depending on needs and goals. Further, the links within may increase awareness of where QI-ROI can be supported, gained or lost, thereby improving alignment of interventions to implementation strategies.

## Conclusion

Our research has answered some important questions regarding what is deemed ROI from QI by healthcare leaders in search of QI programmes that support their strategic goals. Further, our work has synthesized the elements of QI-ROI that support pro-active, responsive, learning, resilient healthcare. Here, we defined the QI-ROI concept as any valued benefit with the four DISS domains. Large-scale QI programmes do require such a comprehensive view of ROI.

## Data Availability

All data pertaining to this work is included in this paper. Any clarification or additional data may be provided by the corresponding author at a reasonable request.
